# Thermal Behaviour of Microgels Composed of Interpenetrating Polymer Networks of Poly(*N*-isopropylacrylamide) and Poly(acrylic acid): A Calorimetric Study

**DOI:** 10.3390/polym14010115

**Published:** 2021-12-29

**Authors:** Silvia Franco, Elena Buratti, Valentina Nigro, Monica Bertoldo, Barbara Ruzicka, Roberta Angelini

**Affiliations:** 1 Department of Basic and Applied Sciences for Engineering, Sapienza University of Rome, Via Antonio Scarpa 14, 00161 Roma, Italy; silvia.franco@uniroma1.it; 2 Institute of Complex Systems (ISC-CNR), Sede Sapienza, Piazzale Aldo Moro 2, 00185 Roma, Italy; elena.buratti@roma1.infn.it (E.B.); valentina.nigro@enea.it (V.N.); 3 Photonics Micro and Nanostructures Laboratory, Fusion and Technologies for Nuclear Safety and Security Department, ENEA C.R. Frascati, V. E. Fermi, 45, 00044 Frascati, Italy; 4 Department of Chemical and Pharmaceutical Sciences, University of Ferrara, 44121 Ferrara, Italy; brtmnc@unife.it; 5 Institute of Organic Synthesis and Photoreactivity (ISOF-CNR), Via Piero Gobetti, 101, 49128 Bologna, Italy; 6 Department of Physics, Sapienza University of Rome, Piazzale Aldo Moro 2, 00185 Roma, Italy

**Keywords:** calorimetry, thermoresponsiveness, microgels, soft colloids, rheology, glass, jamming, poly(*N*-isopropylacrylamide)

## Abstract

Stimuli-responsive microgels have recently attracted great attention in fundamental research as their soft particles can be deformed and compressed at high packing fractions resulting in singular phase behaviours. Moreover, they are also well suited for a wide variety of applications such as drug delivery, tissue engineering, organ-on-chip devices, microlenses fabrication and cultural heritage. Here, thermoresponsive and pH-sensitive cross-linked microgels, composed of interpenetrating polymer networks of poly(*N*-isopropylacrylamide) (PNIPAM) and poly(acrylic acid) (PAAc), are synthesized by a precipitation polymerization method in water and investigated through differential scanning calorimetry in a temperature range across the volume phase transition temperature of PNIPAM microgels. The phase behaviour is studied as a function of heating/cooling rate, concentration, pH and PAAc content. At low concentrations and PAAc contents, the network interpenetration does not affect the transition temperature typical of PNIPAM microgel in agreement with previous studies; on the contrary, we show that it induces a marked decrease at higher concentrations. DSC analysis also reveals an increase of the overall calorimetric enthalpy with increasing concentration and a decrease with increasing PAAc content. These findings are discussed and explained as related to emerging aggregation processes that can be finely controlled by properly changing concentration, PAAc content an pH. A deep analysis of the thermodynamic parameters allows to draw a temperature–concentration state diagram in the investigated concentration range.

## 1. Introduction

The interest in colloidal systems extends from fundamental studies [1] to technological applications [2,3]. Due to their relatively large dimensions with respect to atomic and molecular systems [4,5], they are characterized by lengths and time scales easily accessible with a wide variety of laboratory techniques. Moreover, their effective interactions can be properly tuned either by synthesis and by chemical-physical parameters [6] giving rise to complex phase diagrams [7,8,9,10,11,12]. Among colloidal systems, microgels, particles of almost spherical shape made of crosslinked polymer networks, dispersed in a solvent, are very intriguing systems [1,13]. Particles of size between nanometers and microns, and the possibility of finely tuning their properties by changing the chemical composition during synthesis, open the door to many perspectives. Besides morphological aspects, one of the main advantages of some microgel particles is their ability to respond to environmental stimuli [1]. In particular, depending on the nature of the constituent monomers, microgel particles could manifest high sensitivity to changes of temperature, pH, electric field, ionic strength, solvent and light pulses [14,15,16]. Interestingly, they offer manifold possibilities in several research fields thanks to their polymer/colloid duality [17]. Indeed, the responsiveness of microgels, coupled with their versatility and relatively easy synthesis methods [18], makes them attractive for several applications, such as drug delivery [19,20,21,22,23,24,25], tissue engineering in artificial muscle [21], bone [26] and cartilage fabrications [27,28,29], for organ-on-chip [30] and microlenses [31,32] device development. Moreover, films based on thermo- and pH-sensitive microgels have been developed to obtain three-dimensional macromaterials [33,34,35] and they have been also used to create photonic crystals like structures [36]. Of note, microgels also find applications in cultural heritage conservation for cleaning of modern and ancient paper [37]. Among all the available stimuli-responsive polymer-based microgels, temperature-sensitive ones are the most popular since, by tuning temperature, they can be easily controlled by changing their size, hydrophilicity and softness, reaching high packing fractions [38,39]. Indeed, the most extensively investigated temperature sensitive microgels are those based on poly(*N*-isopropylacrylamide) (PNIPAM) that presents a Lower Critical Solution Temperature (LCST) in water at 305 K. LCST signs the crossover between dominant hydrophilic and hydrophobic interactions between water molecules and PNIPAM polymer [40]. At temperatures below the LCST in fact, water is a good solvent for PNIPAM and polymer chains are hydrated and solvated by water due to strong hydrogen bonds between water and the amide groups (CONH) of PNIPAM. However, the hydrophilic/hydrophobic interactions between water molecules and PNIPAM chains are highly temperature sensitive and when temperature is increased, PNIPAM chains become increasingly hydrophobic [40] undergoing a transition at the LCST, from a coiled and enthalpic favoured structure into an entropically favoured and dense globular one [41,42]. The coil-to-globule transition of PNIPAM polymer characterizes the behaviour of PNIPAM-based microgels that swell at low temperatures, with large numbers of water molecules retained into the crosslinked polymeric structure, and deswell at higher temperatures when water is expelled from the particles giving rise to the typical Volume Phase Transition (VPT) [43,44].

Therefore, to investigate the basic mechanisms of thermoresponsive smart microgels, Differential Scanning Calorimetry (DSC) turns out to be one of the most suitable techniques [45], generally employed to study the thermal transitions of polymeric materials, food and pharmaceutical products, glasses and ceramics, biological macromolecules such as proteins, and in general of soft materials with significant transitions with temperature [46,47,48,49,50,51,52]. Several works have been conducted over the past 20 years, with the aim to investigate, through DSC, the thermal behaviour of hydrogels [53,54,55] and microgels [56,57,58,59]. For instance, the thermal behaviour of PNIPAM-PNIPMAM core shell microgels in D${}_{2}$O solution has been studied by varying the core-shell mass ratio in order to control the mutual influences of the two polymers [60]. Indeed, these particles showed a two-step shrinking behaviour, corresponding to the contributions of the core and the shell, respectively. In the same years, always through DSC, Hoare and Pelton studied the thermal phase transitions of a range of NIPAM-based carboxylic acid-functionalized microgels with well-defined radial and chain functional group distributions [57]. Thermograms displayed a two-peak transition, like in core-shell microgels, induced by the local heterogeneities within the functionalized microgel. In this way, the ratio between the two transition temperatures gives an idea of the radial functional group distribution. Furthermore, PNIPAM microgels with different crosslinker contents were investigated through DSC analysis [56] in order to evaluate the crosslinker contribution, revealing an increase of the transition temperature and a decrease of the overall calorimetric enthalpy with increasing crosslinker concentration. In a further study, the VPT and the fluid-to-glass transition of PNIPAM microgels have been studied through rheological and calorimetric measurements at various concentrations [58]. Always on PNIPAM microgel, the investigation of the volume phase transition has been carried out combining elastic incoherent neutron scattering and differential scanning calorimetry starting from polymer weight concentration of 30% up to dry conditions [59]. They outlined that the VPT emerges from the complex amphiphilic nature of the polymer that is composed by a hydrophobic backbone decorated with side chain groups containing both hydrophilic amide moieties and hydrophobic isopropyl ones. In a very recent work [61] the role of water-polymer interactions on the VPT, the melting and crystallization processes of PNIPAM linear chains and PNIPAM microgels have been deeply investigated. DSC measurements up to 80% of weight concentration were performed to investigate the role of polymer structure and to probe the capability of efficiently confining water. The aging of the samples and the properties of non-deuterated and deuterated microgels were also studied. Of note, colloidal PNIPAM microgels crosslinked with bisacrylamide and with acrylic acid as a comonomer, have been investigated as a function of temperature, pH and ionic strength and then compared with standard PNIPAM [62] displaying different properties. Since the copolymers are temperature-, pH- and electrolyte-sensitive, they offer significant advantages over their homopolymeric analogues having additional “triggering” variables. Moreover, the porous structures of a copolymer microgel of NIPAM and acrylic acid and a poly(acrylic acid) microgel were compared through the measurement of their thermal behaviours [63]. Very recently, microgels functionalized by the introduction of acrylic acid as comonomer or interpenetrating polymer network have been investigated by high-sensitivity differential scanning calorimetry in order to understand their thermoresponsive behaviour [64]. It was found that, in the case of interpenetrating networks, the transition temperature typical of PNIPAM was not notably affected and that the transition cooperativity was reduced at variance with the copolimer network that revealed a highly cooperative thermoresponsive behaviour. In recent years, the investigation of PNIPAM and PNIPAM/PAAc microgels has highly increased as is also demonstrated in the list in Table 1 that reports manuscripts which appeared in the *Polymers* journal in 2021 on similar topics.

At variance with previous studies, here we report an extensive calorimetric study on a microgel composed of Interpenetrating Polymer Networks (IPN) of PNIPAM and poly(acrylic acid) (PAAc), thermo- and pH-sensitive respectively. As previously described, PNIPAM polymer undergoes a reversible coil-to-globule transition around the LCST (305K), while PAAc undergoes a reversible coil-to-globule transition around a pH value close to the pKa ≈ 4.5 that depends on the ionization state of the carboxylic group (COOH). As the pH is increased above the pKa, ionization into H${}^{+}$ + RCOO${}^{-}$ is favoured and the polymer expands into a fully solvated open coil conformation (hydrophilic). However, at low pH, carboxylic group are fully protonated (hydrophobic) and PAAc adopts a more compact globular conformation. The characteristics of the two constituent polymers give rise to an IPN microgel whose VPT can be controlled by properly tuning temperature, pH and the mutual content of PNIPAM and PAAc. This allows to finely control microgel properties through an ad-hoc synthesis procedure. Here we synthesize IPN microgel, by a precipitation polymerization method in water, and we investigate their behaviour through differential scanning calorimetry across the volume phase transition temperature of PNIPAM microgels as a function of heating/cooling rate, concentration, pH and PAAc content. Indeed, a deep analysis of the thermodynamic parameters allows us to draw a temperature–concentration state diagram in the investigated concentration range. To the best of our knowledge, a similar study has never been reported before.

## 2. Materials and Methods

### 2.1. Materials

*N*-isopropylacrylamide (NIPAM) monomer, acrylic acid (AAc), sodium dodecyl sulphate (SDS) (98% purity), potassium persulfate (KPS) (98% purity), ammonium persulfate (APS) (98% purity), N,N,N′,N′-tetramethylethylenediamine (TEMED) (99% purity), ethylenediaminetetraacetic acid (EDTA), NaHCO${}_{3}$, all from Sigma-Aldrich (St. Louis, MO, USA), and the crosslinker N,N′-methylene-bis-acrylamide (BIS), from Eastman Kodak (Rochester, NY, USA), were used. NIPAM monomer and BIS, used as crosslinker, were recrystallized from hexane and methanol respectively, dried under reduced pressure (0.01mmHg) at room temperature and stored at 253K. AAc monomer was purified by distillation (40mmHg, 337K) under nitrogen atmosphere in presence of hydroquinone and stored at 253K. SDS, used as surfactant, KPS and APS, used as initiators, TEMED, a reaction accelerator, EDTA, a chelating agent for purifying dialysis membranes, and NaHCO${}_{3}$, were all used as received. All other solvents were RP grade (Carlo Erba, Cornaredo, Italy) and were used as received. Ultrapure water (resistivity: 18.2MΩ/cm at 298K) was obtained with Sarium^®^ pro Ultrapure water purification Systems, Sartorius Stedim, Göttingen, Germany. A dialysis tubing cellulose membrane, MWCO 14,000 Da, (Sigma-Aldrich) was cleaned before use by washing with running distilled water for 3 h, treated at 343K for 10 min into a solution containing 3.0% NaHCO${}_{3}$ and 0.4% EDTA weight concentration, rinsed in distilled water at 343K for 10min and then in fresh distilled water at room temperature for 2h.

### 2.2. Microgel Synthesis

**Figure 1:**
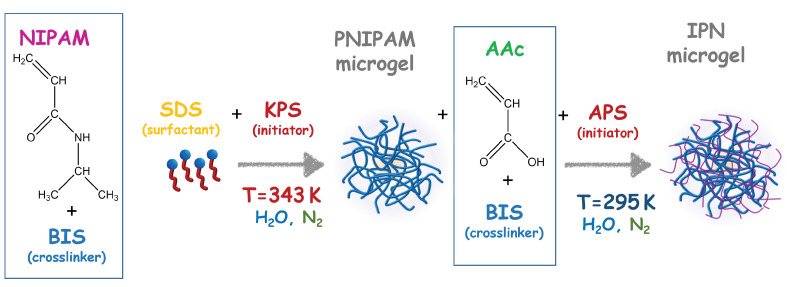
Schematic representation of the synthesis process that involves *N*-isopropylacrylamide (NIPAM) and acrylic acid (AAc) monomers, the crosslinker N,N′-methylene-bis-acrylamide (BIS) and the reagents: sodium dodecyl sulphate (SDS), potassium persulfate (KPS), ammonium persulfate (APS).

IPN microgels were obtained by a sequential free radical polymerization method [66,67] interpenetrating PAAc network in PNIPAM microgels, synthesized by a precipitation polymerization method following the procedure described by Pelton and et al. [13] as illustrated in Figure 1.

In the synthesis, (24.162 ± 0.001)g of NIPAM monomer, (0.4480 ± 0.0001)g of crosslinker BIS and (3.5190 ± 0.0001)g of surfactant SDS were solubilised in 1560mL of ultrapure water and transferred into a 2000mL four-necked jacketed reactor equipped with condenser and mechanical stirrer. Then, the solution, heated at (343 ± 1)K, was deoxygenated by purging with nitrogen for 1h. The addiction of (1.0376 ± 0.0001) g of KPS, dissolved in 20mL of deoxygenated water, allows the polymerization to begin and it is stopped after 5 h. The resultant PNIPAM microgel was purified by dialysis against distilled water for two weeks, changing water frequently. Through gravimetric measurements, the final weight concentration C${}_{w}$ of PNIPAM micro-particles was determined ∼1%. In the second step of the two-step process for the synthesis of IPN microgels, (140.08 ± 0.01) g of the recovered PNIPAM dispersion (C${}_{w}$ = 1.06%) was diluted with ultrapure water up to a volume of 1260mL into a 2000 mL four-necked jacketed reactor, kept at (295 ± 1)K by circulating water. Then, 5mL of AAc monomer and (1.1080 ± 0.0001)g of BIS crosslinker were added to the dispersion and the mixture was deoxygenated by bubbling nitrogen inside for 1h. 0.56mL of accelerator TEMED were poured and the polymerisation was started with (0.4447 ± 0.0001)g of initiator APS. The polymerisation reaction, carried out at pH 3, was stopped by exposing to air and PAAc content, depending on the time at which the reaction is stopped. The resultant IPN was purified by dialysis against distilled water with frequent water changes for two weeks, and then lyophilised and redispersed in water to prepare samples at weight concentration C${}_{w}$ = 1.0%. The PAAc weight concentrations (C${}_{PAAc}$) of the synthesized IPN samples were determined by combination of elemental and ${}^{1}$H-NMR analysis as described in reference [68], they were C${}_{PAAc}$ = 2.7%, 4.5%, 15.4% and 24.6%. Samples at low concentrations (lower than C${}_{w}$ = 1.0%) were obtained by dilution with distilled water from the same stock suspension at C${}_{w}$ = 1.0% and adjusted at pH = 5.5. Moreover, samples at higher concentrations were obtained by evaporation from the sample C${}_{w}$ = 1.0%. Samples at fixed C${}_{w}$ and different pH were obtained by adding suitable quantities of HCl solution (in the range 0.1–1M) for samples at acid pH or NaOH (in the range 0.1–10M) for those at basic pH.

### 2.3. Differential Scanning Calorimetric Measurements

**Figure 2:**
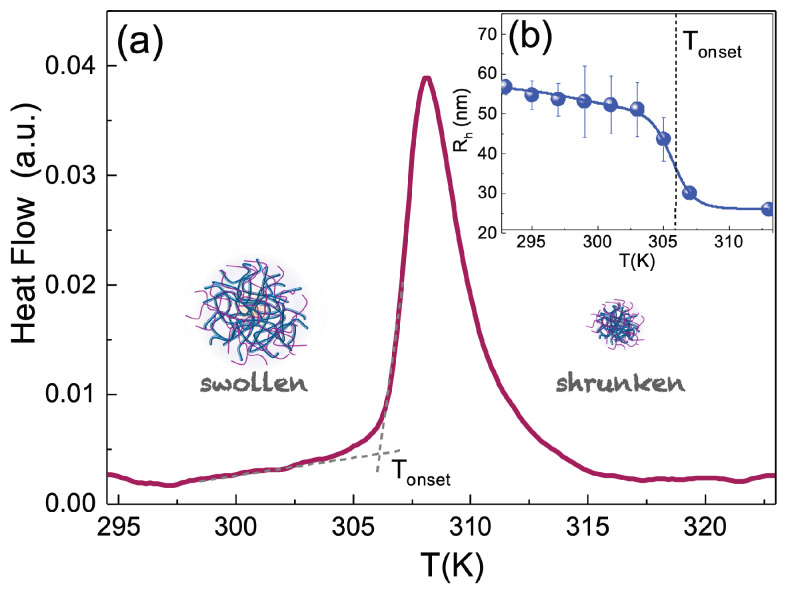
(**a**) DSC thermogram of an IPN microgel at C${}_{PAAc}$ = 2.7%, C${}_{w}$ = 0.9%, pH 5.5 and heating rate 10K/min. The onset temperature is indicated by the intersection of the dashed lines. (**b**) Hydrodynamic radius $R_{h}$, for the same IPN microgel, as a function of temperature measured in dilute conditions (C${}_{w}$ = 0.01%). The dashed line indicates the onset temperature obtained from DSC measurements.

In order to locate the volume phase transition typical of microgels, differential scanning calorimetric measurements were carried out with a Diamond Perkin-Elmer power compensation calorimeter equipped with the data acquisition software Pyris. Purge gas (nitrogen) was let through the DSC cell with a flow rate of 20mL/min. Temperature was calibrated with indium zinc and distilled water and the indium latent heat of melting was used for calibrating heat flow. At constant pressure all heat supplied to the system dQ/dt helps to increase its internal energy and is related to the specific heat of the system, therefore [69,70]:

(1) $$\frac{dH}{dt}=\frac{dQ}{dt}=mc_{p}dT$$

where *m* is the mass of the sample, $c_{p}$ the specific heat and dT the differential temperature variation. Thus, integrating, the area is [69]:

(2) $$\int_{T_{1}}^{T_{2}}\frac{dQ}{dt}dT=mr\int_{T_{1}}^{T_{2}}c_{p}\left(T\right)dT$$

where $r=\frac{dT}{dt}$ is the heating/cooling rate. The thermodynamic quantity dQ/dt, i.e., the thermal power is measured with an instrument sensitivity of 0.2mW Sealed standard aluminium sample pans with a volume of 10 μL and 50 μL were used as cells and references. Samples, initially at room temperature, were brought to T = 278K and held in this condition for 3 min, then were heated to T = 323K across their volume phase transition at a rate of 10K/min. An example of thermograms, i.e., the heat flow dH/dt versus temperature T, is displayed in Figure 2a for an IPN microgel at $C_{PAAc}$ = 2.7% and C${}_{w}$ = 0.9%. It represents the typical coil-to-globule transition associated to the thermosensitive PNIPAM microgel and it is reversible, as verified by cooling down the sample.

All measurements were repeated several times in order to verify their reproducibility and to evaluate their uncertainty, all transitions were found to be reproducible and reversible. IPN microgels with four different PAAc contents, $C_{PAAc}$ = 2.7%, 4.5%, 15.4% and 24.6%, were investigated at different weight concentrations from C${}_{w}$ = 0.3% to 10.0%. The characteristic parameters of the different IPN microgels are reported in Table 2.

### 2.4. Dynamic Light Scattering Measurements

The particle hydrodynamic radius $R_{h}$ of IPN microgels was measured through Dynamic Light Scattering (DLS) as a function of temperature T. An optical setup based on a solid state laser (100mW) with monochromatic wavelength λ = 642nm and polarised beam was used to probe IPN microgels suspensions in dilute regime. Measurements were performed at a scattering angle θ = 90${}^{\circ }$ that corresponds to a scattering vector Q = (4πn/λ) sin(θ/2) = 0.018$\;{\mathrm{nm}}^{-1}$. The hydrodynamic radii were obtained through the Stokes–Einstein relation: $R_{h}$ = k${}_{B}$T/6$\pi \eta_{s}$ D${}_{t}$ where k${}_{B}$ is the Boltzmann constant, $\eta_{s}$ the viscosity of the solvent, namely water, at the measured temperature and D${}_{t}$ the translational diffusion coefficient related to the relaxation time τ through the relation: τ = 1/(Q${}^{2}$ D${}_{t}$). The relaxation time was obtained by fitting the autocorrelation function of scattered intensity through the Kohlrausch–William–Watts expression [71,72], $g_{2}\left(Q,t\right)$ = $1+b{\left[exp\left(-{\left(t/\tau \right)}^{\beta }\right)\right]}^{2}$, with the stretching exponent β providing the deviation from the single exponential. Measurements were performed as a function of temperature at a heating rate 0.1$\;K/\min^{-1}$. The samples were held for 5min at each temperature before measurement. An example of the hydrodynamic radius behaviour for a chosen IPN microgel is reported in Figure 2b.

To evaluate the capability of an IPN microgel particle to shrink, the swelling ratio [73] can be employed, it is defined as:

(3) $$\alpha =\frac{R_{h}^{swollen}}{R_{h}^{shrunken}}=\frac{R_{h(293K)}}{R_{h(313K)}}$$

where $R_{h}^{swollen}$ and $R_{h}^{shrunken}$ are the hydrodynamic radii in the swollen and in the shrunken state. $R_{h}^{swollen}$ and $R_{h}^{shrunken}$ were chosen as the values at T = 293K and T= 313K, respectively. α values for the investigated IPN microgels are reported in Table 2: α decreases with increasing PAAc content, indicating a reduced capability of microgel to shrink as deeper discussed later.

## 3. Results and Discussion

In order to evaluate the thermal behaviour of IPN microgels across the volume phase transition as a function of weight concentration C${}_{w}$, PAAc content and pH, we performed DSC measurements at four PAAc contents, different weight concentrations and seven different pH, in the temperature range (278–323)K, at heating rate 10K/min.

Figure 2a shows, as an example, the thermogram of an IPN microgel with C${}_{PAAc}$ = 2.7% and C${}_{w}$ = 2.8% and pH 5.5. A peak of endothermic nature is observed in correspondence with the volume phase transition temperature, typical of the thermosensitive PNIPAM (T ≈ 305K), which indicates the transition from the swollen to the shrunken state of particles and indicates that, during heating, particles absorb energy to reach a new thermoreversible state characterized by a reduced size, changed mutual interactions and spatial configurations. The onset temperature, T${}_{on}$, indicated in Figure 2a as the intersection between two dashed lines, is in agreement with that found through DLS in Figure 2b, where the hydrodynamic radius $R_{h}$, measured in very dilute conditions (C${}_{w}$ = 0.01%), shows the transition from a swollen to a shrunken state. At this concentration and PAAc content, the transition temperature of IPN microgels does not deviate remarkably from that of PNIPAM microgels [64].

### 3.1. Effect of Heating Rate

**Figure 3:**
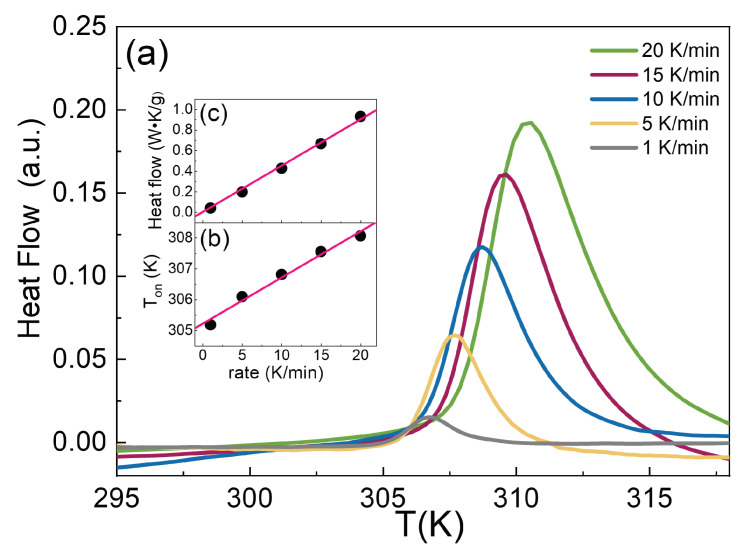
(**a**) DSC thermograms of IPN microgel at C${}_{PAAc}$ = 2.7%, C${}_{w}$ = 2.8%, pH 5.5 and different heating rates. (**b**) Onset temperature and (**c**) Normalized heat flow as a function of heating rate. Lines are linear fits to data.

With the aim of understanding the effect of heating rate on the VPT temperature, Figure 3a shows the thermograms of IPN microgel with C${}_{PAAc}$ = 2.7% and C${}_{w}$ = 2.8% at different heating rates from 1 to 20K/min. The heating rate greatly affects the volume phase transition temperature ranging from 305K to 308K. This indicates that the heating rates are still high enough for the microgels to undergo a quasi-equilibrium phase transition and that the process of particle deswelling has extremely slow kinetics.

The onset temperatures are plotted as a function of the scan rate in Figure 3b and by extrapolating the plot to zero scan rate, one obtains the adiabatic quasi-equilibrium phase transition temperature of 305.2K. Therefore, slower heating rates favour the occurrence of the transition at lower temperatures; on the other hand, by increasing heating rate, the transition temperature is shifted to higher values. This can be explained considering that, when temperature is gradually changed, microgel particles have enough time to reach the new final state, while with a rapid change of T, they require more time to rearrange and the transition is shifted to higher temperatures. These results aid to reconcile different values for the VPT temperatures reported in several works, also in the case of the parental PNIPAM microgel where measurements were carried out with different techniques and heating rates. Figure 3a also indicates that the endothermic peaks, whose area below represents the energy required by the microgels to undergo the transition, are less pronounced at lower heating rates. In fact, at low heating rates, microgels have enough time to expel most of the solvent and less energy is required to achieve the necessary volume changes. On the contrary, at faster rates, the swelling kinetics are slower than the scan rate, the system is out of equilibrium and more heat is necessary for microgel deswelling. Normalized heat flow by the factor (temperature/mass) is plotted as a function of the scan rate in Figure 3c. By extrapolating the plot to zero scan rate, one obtains the quasi-equilibrium enthalpy variation normalized by the mass of 2.26J/g.

### 3.2. Effect of Weight Concentration

**Figure 4:**
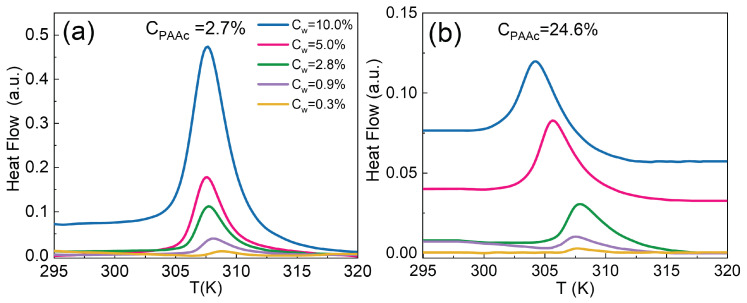
DSC thermograms for IPN microgels (**a**) at C${}_{PAAc}$ = 2.7% and (**b**) C${}_{PAAc}$ = 24.6%, pH 5.5, at different concentrations and fixed heating rate 10K/min.

To understand how weight concentration affects the volume phase transition of IPN microgels we performed DSC measurements in the range 0.3% to 10.0%. Figure 4a,b show an example of thermograms for two IPN microgels, at low (C${}_{PAAc}$ = 2.7%) and high (C${}_{PAAc}$ = 24.6%) PAAc contents, at different weight concentrations and at fixed heating rate of 10K/min. In both cases, with increasing weight concentration, the transition peak becomes higher and sharper since more particles require a greater amount of energy to undergo the VPT. Moreover, at the lowest PAAc content, the curves show the same plateau before and after the VPT, at variance with the highest concentration C${}_{w}$ = 10.0% where a step is observed.

This latter behaviour is typical of a liquid-to-glass transition [74]. Instead, for IPN with C${}_{PAAc}$ = 24.6%, already at concentration C${}_{w}$ = 0.9%, the baseline variation appears, becoming even more evident at the highest concentrations (here thermograms are properly shifted upwards to better visualize the differences) suggesting the presence of a glass transition that accompanies the typical VPT of microgels at a much lower C${}_{w}$ with respect to IPN with C${}_{PAAc}$ = 2.7%. These results provide a calorimetric interpretation of recent findings obtained through rheological measurements [75], DLS and X-ray photon correlation spectroscopy [76] carried out on the same sample where, by studying respectively the stress flow curves and the normalized intensity autocorrelation functions $g_{2}\left(q,t\right)-1$, we distinguished the transition from liquid to glassy state at a concentration around C${}_{w}$ = (0.6–0.7)%. Overall, for the highest PAAc content, a further feature is observed: the transition temperature is shifted at lower values at higher concentrations as will be better discussed later.

### 3.3. Effect of pH

**Figure 5:**
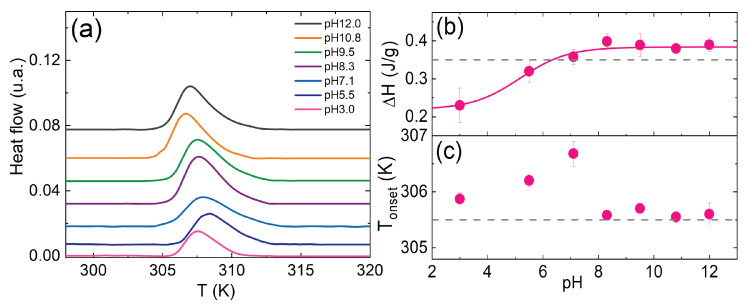
(**a**) DSC thermograms for an IPN microgels with C${}_{PAAc}$ = 15.4%, C${}_{w}$ = 1.0% at different pH and at heating rate 10K/min. (**b**) Area normalized by mass as a function of pH (pink line is a guide to eyes) and (**c**) Onset temperature. Dashed lines represent the values of pure PNIPAM.

In order to evaluate the effect of pH, we investigated the thermal behaviour of an IPN microgel with PAAc content 15.4% and C${}_{w}$ = 1.0% at pH 3.0, 5.5, 7.1, 8.3, 9.5, 10.8, 12.0 and at heating rate 10K/min, as shown in Figure 5a. Thermograms are properly shifted upwards to better visualize the differences. As already reported in the introduction, the pH-sensitive PAAc network is characterized by a coil-to-globule transition around pH 5 due to the ionization of the carboxylic group into H${}^{+}$ + RCOO${}^{-}$. Below this pH it assumes a compact globular conformation while, by increasing pH, the fraction of ionized groups RCOO${}^{-}$ increases and PAAc expands into a coil conformation [77,78]. Therefore, tuning pH allows to tune water-particle and particle-particle interactions: at high pH in fact, the coil conformation of PAAc chains provides a more pronounced dangling chain structure to the IPN microgel particles. Moreover the COOH groups of PAAc are dissociated in COO${}^{-}$ and H${}^{+}$ preventing H-bonding between different particles, but favouring like-charge attraction that results from counterion fluctuation due to the formation of temporary dipoles. This is evident from the growth of the structural relaxation time across the VPT for IPN microgel at C${}_{PAAc}$ = 19.2% and C${}_{w}$ = 0.9% which indicates a slowing down of the dynamics and the formation of aggregates [73]. Hence, at these pH, interactions among particles and aggregation are favoured.

**Figure 6:**
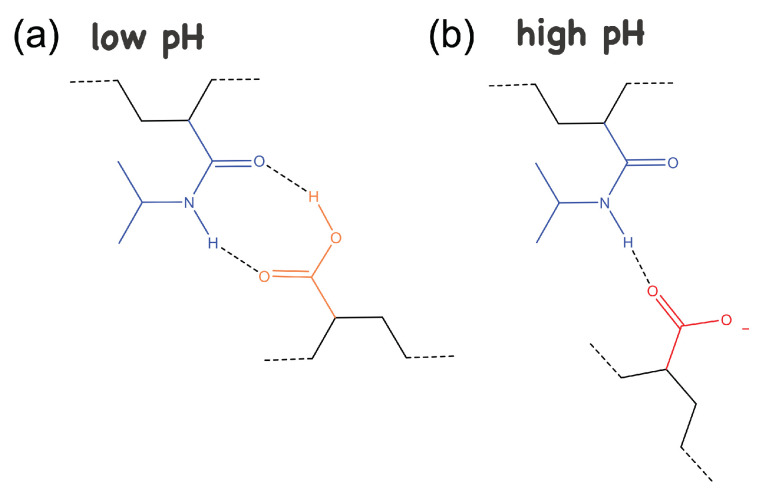
Sketch of the PNIPAM-PAAc interactions: (**a**) at low pH, when the carboxylic groups (COOH, in orange) of AAc are protonated, the formation of complexions with the amide groups (CONH, in blue) of NIPAM occurs, through two hydrogen bonds (dashed black lines); (**b**) at high pH, the AAc carboxylic groups are deprotonated (COO${}^{-}$, in red) and a single hydrogen bond may form with NIPAM.

On the contrary, at low pH, PAAc is insoluble in water and hydrogen bonds between its carboxylic (COOH) groups and the isopropyl (CONH) groups of PNIPAM are formed (Figure 6a), with a consequent globule configuration that gives a more compact shape to the IPN particles and makes them more hydrophobic and with reduced capability of interaction both with water and other particles. This trend is reflected clearly in the behaviour of the area under the peak normalized by the sample mass, ΔH (J/g), (Figure 5b), and of the onset temperature, T${}_{on}$, (Figure 5c). The area under the peak normalized by the sample mass, namely the normalized enthalpy variation, that is related to the dehydration process of the isopropylic groups of PNIPAM, increases with increasing pH and shows a change of slope around pH 5 (that reminds the typical PAAc titration curve), indicating that less energy is required to undergo the transition at low values.

In fact, at low pH, most of the carboxylic groups (COOH) of PAAc are protonated and a complexation with the amide groups (CONH) of the NIPAM repeating units through hydrogen bonds occurs, as shown in Figure 6a. These repeating units involved in the complexation probably do not contribute to the transition, which then shows low values of enthalpy, due to the small amount of NIPAM units undergoing the process. However, increasing the pH, the amount of deprotonated carboxylic acid increases and at high values the hydrogen bonds with NIPAM no longer happen, due to repulsive interactions (Figure 6b). Thus, the complexation is broken and almost all the NIPAM repeating units are involved in the transition, as shown by the value of ΔH very similar to the one of pure PNIPAM (dashed line in the figure) ΔH = 0.35 J/g. A similar behaviour is observed in Figure 5c for T${}_{on}$ that overlaps with the VPT temperature of PNIPAM (305.5K) above pH 8, confirming that PAAc no longer affect the PNIPAM transition thanks to the break up of the hydrogen bonds of the complexes between NIPAM and AAc. However, decreasing the pH, an increase of T${}_{on}$ is observed around 7, followed by a further slight decrease toward lower values, probably due to the complexation between NIPAM and AAc repeating units, which leads to an anticipation of the VPT. At these pH, the completely open PAAc structure with PAAc dangling chains favours particle aggregation, resulting in an overall decrease of the transition temperature, as also found varying C${}_{w}$.

### 3.4. Effect of PAAc Content

**Figure 7:**
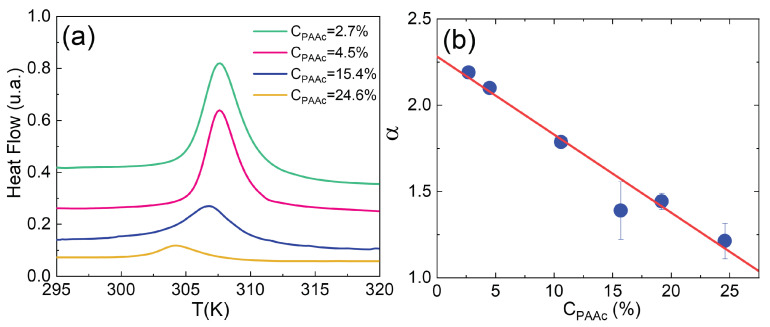
(**a**) DSC thermograms for IPN microgels with different PAAc contents at C${}_{w}$ = 10.0%, pH 5.5 and at heating rate of 10 K/min. (**b**) Swelling ratio from Equation (3) for IPN microgels at different PAAc contents.

With the aim of evaluating the effect of the second PAAc polymer network on thermal behaviour, we report in Figure 7a the heat flow of IPN microgels with different PAAc contents, 2.7%, 4.5%, 15.4% and 24.6%, at C${}_{w}$ = 10.0%, pH 5.5 and at heating rate 10K/min. The area under the peak normalized by the sample mass decreases with increasing PAAc content, indicating that less energy is required to undergo the transition. This trend suggests that the endothermic transitions strongly depend on the presence of the second polymeric network of PAAc that hinders the contraction of the particles both because it constitutes a topological constraint [73] and also due to hydrogen bonds of PAAc with H${}_{2}$O, since at pH 5.5, above the critical pH threshold (pK${}_{a}\approx$ 4.5), polyacrylic acid becomes hydrophilic [64,79,80,81].

Furthermore, besides the steric hindrance of the PAAc chains, a nontrivial role is played by the crosslinker BIS, which, as shown in Table 2, is greater for the highest PAAc content. These findings agree with those of an interesting work by Grinberg and coworkers [64] where the volume phase transition of an interpenetrated microgel of PNIPAM and PAAc differs notably from both P(NIPAM-*co*-PAAc) and pure PNIPAM microgel showing a lower enthalpy, a larger transition width and an onset temperature shifted to higher values. They attribute this behaviour to the fact that interpenetration of the second network reduces the transition cooperativity. These results are also in agreement with a recent work [82] that compares the thermoresponsive behaviour of a double hydrogel network (DN) with that of a single hydogel network (SN). DN hydrogels display smaller enthalpy than SN, confirming that the presence of a second hydrophilic network (PAAc in our case) has a noticeable impact on thermal sensitivity. These arguments are also supported by the behaviour of the swelling ratio α, defined through Equation (3) and shown in Figure 7b, that highlights a reduced shrinking capability for microgels with higher PAAc content.

**Figure 8:**
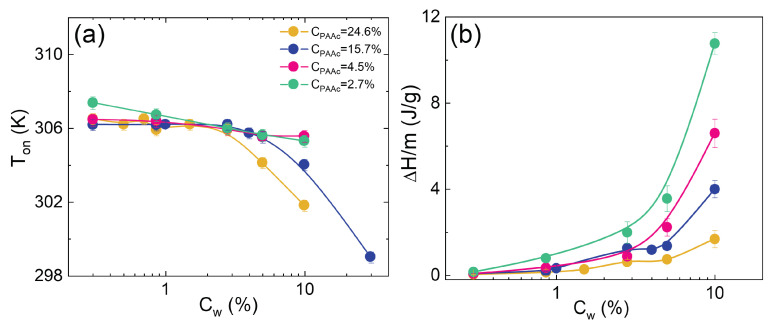
(**a**) T${}_{onset}$ and (**b**) ΔH/m versus C${}_{w}$ for IPN microgels with different PAAc contents at pH 5.5.

In Figure 8a the onset temperatures are reported as a function of concentration at different PAAc contents: C${}_{PAAc}$ = 2.7%, 4.5%, 15.4% and 24.6%. For all the samples an almost constant behaviour is observed at low concentrations, followed by a decrease as concentration increases. In particular, for samples C${}_{PAAc}$ = 2.7% and 4.5%, T${}_{onset}$ slightly decreases in the investigated concentration range, while for microgels with higher PAAc contents it systematically bends at increasing concentrations with a deflection point that depends on PAAc content. These results can be interpreted considering that, at low PAAc content and weight concentration, T${}_{onset}$ corresponds to the typical LCST of PNIPAM while with increasing PAAc content and microgel concentrations the VPT is accompanied by the presence of a transition towards an arrested state, as highlighted in the description of Figure 4. Such a transition is promoted for microgels at high C${}_{PAAc}$ indicating that, at high C${}_{w}$, PAAc contributes to a shift of the transition temperature at lower T. In fact, at this pH (pH 5.5), the ionization of the carboxylic group into H${}^{+}$ + RCOO${}^{-}$ occurs, the fraction of deprotonated moieties (COO${}^{-}$) is not negligible and the polymer expands towards a solvated open coil conformation [79,83] where interparticle interactions and aggregation are favoured due to like-charge attraction [73]. This tendency is stronger at higher PAAc contents. Figure 8b shows the enthalpy normalized by the sample mass m as a function of concentration. At fixed PAAc content, the area under the peak becomes greater with increasing weight concentration and this is explained considering that the system contains a large number of microgel particles, as anticipated qualitatively in Figure 3.

Moreover, a systematic decrease of energy is observed with increasing PAAc, confirming that microgels have a reduced deswelling capability when a second network is interpenetrated. An important feature emerges from Figure 8b: curves have a different shape by varying PAAc, for high PAAc contents, a rather linear increase at low concentrations is followed by a change in slope which generally occurs earlier in C${}_{w}$ with increasing C${}_{PAAc}$. Conceptually, the change of trend of enthalpy could be the hallmark of the existence of different phases in the system.

**Figure 9:**
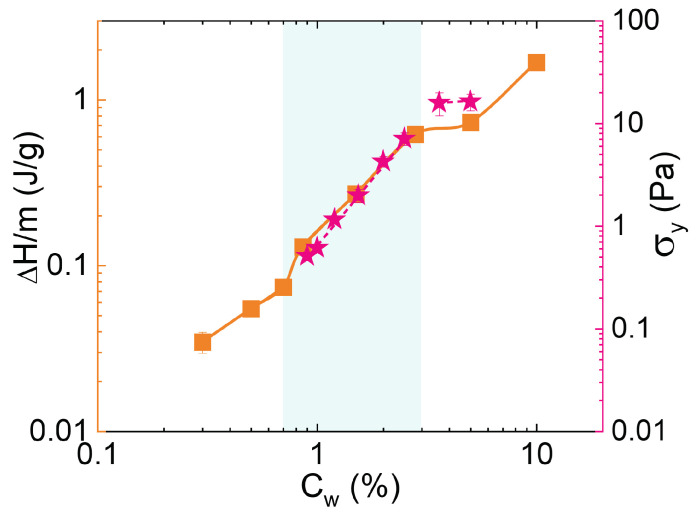
Comparison of ΔH/m versus concentration (orange squares, left axis) with the yield stress obtained from the rheological measurements (pink stars, right axis) reported in reference [75] for an IPN at C${}_{PAAc}$ = 24.6% and pH 5.5.

To better highlight phase changes, in Figure 9 the plot of ΔH/m versus C${}_{w}$ for the highest PAAc content C${}_{PAAc}$ = 24.6% is shown. In particular, three different regions are distinguished: C${}_{w}≲$ 0.7%, 0.7% ≲ C${}_{w}$≲ 3.0% and C${}_{w}≳$ 3.0%. A comparison with the apparent yield stress, obtained from rheological measurements discussed in reference [75], highlights the perfect agreement and provides a calorimetric characterization of three different kind of transition: between two viscous liquids at low microgel concentrations, a liquid–glass transition at intermediate concentrations and a transition between a glass and a jamming state at higher C${}_{w}$.

**Figure 10:**
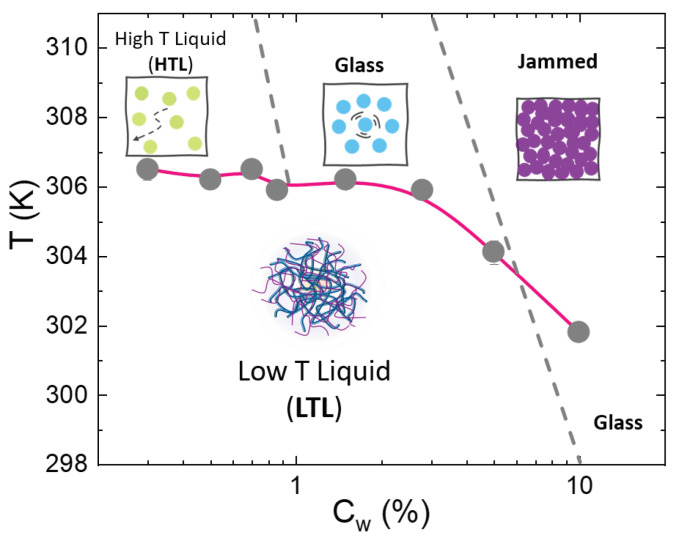
T-C${}_{w}$ Phase diagram of IPN microgel suspensions with PAAc content 24.6% and pH 5.5. The onset transition temperatures obtained with DSC measurements at a heating rate r = 10 K/min are plotted as a function of concentration.

On the basis of the DSC analysis, we report in Figure 10 a preliminary temperature–concentration phase diagram for an IPN microgel with C${}_{PAAc}$ = 24.6%. *Below the VPT*, at low temperatures and concentrations, particles are free to diffuse and interactions are unfavoured so that microgel suspensions behave like viscous liquids: we call this phase “low temperature liquid (LTL)”. With increasing concentration, the motion of the particles slows down and the system shows a glass state characterized by a caging regime in which particles rattle within the cage of their nearest neighbours. *At the VPT*, for low concentrations, the transition temperature overlaps to the LCST of PNIPAM while at increasing concentrations the VPT is accompanied by the formation of an arrested state giving rise to a downward deflection of the transition line. *Above the VPT*, when microgel particles collapse in the shrunken state, three different states are distinguished depending on concentration. (i) In the low concentration regime, the collapse of PNIPAM networks enhances PAAc exposure, however microgels suspensions are too diluted to form arrested states and still behave like a viscous liquid, we call it “high temperature liquid” (HTL). (ii) At increasing concentrations, the system exhibits a glassy state, here interactions among particles are favoured since the collapse of the thermosensitive PNIPAM promotes the exposure of PAAc and the like-charge attractions [73] resulting in the formation of an attractive glass [75]. (iii) Finally, when particles are packed at even greater concentrations, due their deformability, they interpenetrate each other, reaching a jammed state. These findings complement rheological measurements reported in ref. [75] and DLS and X-ray photon correlation spectroscopy in ref. [76], allowing to draw a T-C${}_{w}$ phase diagram. The dividing lines between different states have been tentatively drawn as diagonal dashed lines considering that the transition concentration increases with decreasing temperature.

Finally, although the main phenomenology is preserved, the obtained results depends on PAAc content, in particular IPN microgels at lower PAAc reach glass or jammed states at higher concentrations, even outside the range investigated in this work.

## 4. Conclusions

In this work, we deeply investigated, through DSC, the thermal behaviour of microgels composed of interpenetrated polymer networks of poly(*N*-isopropylacrylamide) and poly(acrylic acid) at different heating rates, weight concentrations, pH and PAAc contents. The adiabatic quasi equilibrium phase transition temperature and enthalpy variation were obtained from heating rate investigation at different concentrations and PAAc contents. Two different scenarios appear: in dilute conditions and at low PAAc contents, the transition temperature, typical of PNIPAM microgels, is not significantly affected from interpenetration of PAAc network, while for high concentrations and PAAc, a marked decrease, attributed to the occurrence of a liquid-to-glass transition, is observed. Moreover, DSC analysis also revealed an increase of the overall calorimetric enthalpy with increasing concentration and a decrease with increasing PAAc content, respectively caused by emerging aggregation and by hindering of the contraction of particles due to the topological constraints of the second network and to complexation of PNIPAM. In fact, at the investigated pH 5.5, the PNIPAM contribution to enthalpy transition is reduced due to complexation of the PNIPAM amide groups with the carboxylic PAAc groups: the higher the PAAc content, the greater the inhibition of PNIPAM transition. This complexation is enhanced at low pH values and correspondingly, the enthalpy of transition decreases with decreasing pH. Based on this DSC study, a temperature–concentration state diagram for IPN microgel composed of PNIPAM and PAAc was drawn for the first time. As expected from the complexity and tunability of the system, different states are present and this study opens the way for further investigations at increasing sample concentration. Moreover, the use of complementary techniques, such as small-angle X-ray scattering or small-angle neutron scattering, will permit to better elucidate the structure of the different states proposed in this paper. Therefore, the study clearly reveals that the interpenetration of PAAc into PNIPAM affects both the thermodynamics and the kinetics of the system. Furthermore, the correlation between the observed thermal behaviour and the rheological properties clearly indicates the possibility to trigger the arrest of the system by the temperature, once the composition of the IPN in terms of PAAc content and concentration of particles in the dispersion have been optimized ad hoc. Fine control of these microgels is fundamental also for their numerous technological applications in different fields such as drug delivery, tissue engineering, organ-on-chip devices, cultural heritage and microlens fabrication.

## Figures and Tables

**Table 1:** Table of the articles published in *Polymers* in 2021 about PNIPAM based microgels.

Reference	Investigated System	Highlighted Topic	Investigation Techniques
Sennato et al. [16]	PNIPAM microgels	electrostatic behaviour	DLS, TEM, AFM, electrophoresis, viscosimetry
Annegarn et al. [18]	P(NIPAM-*co*-APMH) microgels	electrostatic behaviour	DLS, AFM, H-NMR
Nasimova et al. [34]	Macromaterials of PNIPAM-PAAc IPN microgels	macromaterial properties	FTIR, computer simulations
Buratti et al. [35]	Films of PNIPAM-PAAc IPN microgels	layer characterization	DLS, H-NMR, AFM contact angle
Nigro et al. [65]	PNIPAM-PAAc IPN microgels	physical behaviour	DLS, SANS, Raman, rheology, electrophoresis

**Table 2:** Characteristic parameters of IPN microgels at different PAAc contents at pH 5.5. $R_{h}$ is the particle hydrodynamic radius measured through DLS at T = 293K and T = 313K at weight concentration C${}_{w}$ = 0.01%, α is the swelling ratio defined in the text through Equation (3), C${}_{PAAc}$, C${}_{PNIPAM}$ and C${}_{BIS}$ are respectively the contents of PAAc, PNIPAM and crosslinker BIS within particles measured through elemental and ${}^{1}$H-NMR analysis [68].

$C_{\mathrm{PAAc}}\;\left(\%\right)$	$R_{h(293K)}\;\left(\mathrm{nm}\right)$	$R_{h(313K)}\;\left(\mathrm{nm}\right)$	α	$C_{\mathrm{PNIPAM}}\;\left(\%\right)$	$C_{\mathrm{BIS}}\;\left(\%\right)$
2.7	57±2	26±1	2.19	94.5	2.8
4.5	66±2	31±1	2.13	91.5	4.0
10.6	93±1	52±1	1.79	88.3	1.1
15.4	120±1	88±1	1.36	79.2	5.4
24.6	159±6	130±3	1.22	67.7	7.7

## Data Availability

The data presented in this study are available on request from the corresponding author.
